# Nurse Engagement in the Hospital Setting: An Analytical Cross-Sectional Multicentre Study With a 4-Year Time Series

**DOI:** 10.1155/jonm/5730405

**Published:** 2025-10-07

**Authors:** Asta Heikkilä, Tarja Kvist, Kristiina Junttila, Pirjo Kaakinen, Outi Kanste, Marja Kaunonen, Tiina Kortteisto, Tiia Rissanen, Susanne Salmela, Tarja Tervo-Heikkinen, Krista Jokiniemi

**Affiliations:** ^1^Department of Nursing Science, University of Turku, Turku, Finland; ^2^Turku University Hospital, The Wellbeing Services County of Southwest Finland, Turku, Finland; ^3^Department of Nursing Science, Faculty of Health Sciences, University of Eastern Finland, Kuopio, Finland; ^4^Nursing Research Center, Helsinki University Hospital, Helsinki, Finland; ^5^University of Helsinki, Helsinki, Finland; ^6^Research Unit of Health Sciences and Technology, University of Oulu, Oulu, Finland; ^7^Faculty of Social Sciences, Health Sciences, Tampere University, Tampere, Finland; ^8^Tampere University Hospital, The Wellbeing Services County of Pirkanmaa, Tampere, Finland; ^9^Department of Biostatistics, University of Turku, Turku, Finland; ^10^The Competence Development Unit, Wellbeing Services County of Ostrobothnia, Vaasa, Finland; ^11^Customer Relationships and Quality, Kuopio University Hospital, Wellbeing Services County of North Savo, Kuopio, Finland

**Keywords:** hospital, nurse, nurse engagement, time series study

## Abstract

**Aim:**

To describe and explain the level of nurse engagement in the period 2019–2022 and identify related background variables and drivers of nurse engagement in hospital settings.

**Design:**

An analytical cross-sectional multicentre study with a 4-year time series.

**Methods:**

Data were collected annually from 2019 to 2022 from 24,653 nurses (staff nurses, midwives, and assistant nurse managers) in Finnish hospitals (*n* = 9) using a modified version of the Nurse Engagement Survey and analysed statistically. The STROBE checklist was used as the reporting guideline.

**Results:**

Nurse engagement varied over the study period, with 10.0%–4.2% of nurses being engaged, 33.1%–17.0% content, 31.4%–28.8% ambivalent, and 25.5%–50.0% disengaged. The proportion of disengaged nurses increased, while the proportions of content and engaged nurses decreased over the 4 years. All background variables and drivers of nurse engagement were statistically significant in relation to the level of nurse engagement.

**Conclusion:**

This study produced novel findings on nurse engagement and related factors in a hospital setting, based on unique, national-level data collected over 4 years. The results raise concerns considering the attractiveness of the nursing profession and nurse retention. The study provides insights for nurse leaders to strengthen leadership practices and create more engaging work environments. To support nurse engagement, healthcare organisations should routinely assess the engagement of their nursing workforce and prioritise leadership strategies aimed at attracting, retaining, and enhancing nurse engagement.

**Implications for Nursing Management:**

It is important that nurse leaders promote nurse engagement throughout nurses' careers and recognise nurses of all generations, as nurse engagement is a key factor in staff retainment and impacts directly on the quality and outcomes of patient care. To keep nurses engaged, nurse leaders and managers should keep patient care manageable and also offer other tasks that match the nurses' interests and career opportunities.

## 1. Introduction

Engagement is typically defined as work engagement or employee engagement. However, it is important to recognise the distinction between different types of engagement. Work engagement is characterised as being vigorous (willingness to invest effort in one's work), dedicated (involvement) and absorbed (concentration) in one's work, while employee engagement is defined as a positive and active state that is related to work and to which the employee's cognition, emotions, and energy are directed, but it is job satisfaction that precedes employee engagement and not the other way around [1]. In this study, nurse engagement contains elements of both work engagement and employee engagement. An engaged nurse “should be inspired by his or her hospital, [be] willing to invest discretionary effort, [be] likely to recommend the employer, and [should be] planning to remain with the hospital for the foreseeable future” [[2], p. 15].

Nurse engagement is important because it directly impacts the quality of patient care [3, 4], which in turn has a positive impact on patient satisfaction with nursing care [5]. Engaged nurses are also more committed, motivated, and satisfied in their roles [6, 7]. Nurse engagement is positively related to job satisfaction, organisational productivity and performance, and a more positive work environment, while it is negatively correlated with nurses' intention to leave their work [5, 8–10]. Decisions to leave the profession are shown to evolve gradually over time and are based on persistent disappointments and a constant mismatch between expectations and reality [11].

To prevent nurses from leaving the profession and to enhance their engagement, it is important to balance job demands with the satisfaction derived from adequate resources required, a supportive environment, and performance feedback [12]. However, nurse engagement is hindered by short work contracts, a heavy workload, and generational differences in values [6, 7]. Therefore, nurse managers and leaders play a key role in promoting nurse engagement [13].

Previous research has demonstrated the stability of nurse engagement [14]. Stability appears to depend on the time period over which it is measured, as results indicate that engagement fluctuates over short work periods [15]. However, despite these brief, temporary variations, engagement tends to return to its usual level over longer periods, and engagement appears to maintain a very stable state [14]. According to a review by Aungsuroch et al. [3], nurse engagement is influenced by individual, organisational, and job- and role-related factors in addition to work–life balance and work environment. Among the organisational-related factors, earlier studies have identified aspects such as leadership style [13, 16], supervisors' support and feedback [17, 18], the organisation's readiness to follow nurse suggestions for performance improvement, a manager's responsiveness and recognition, nurses' perception of autonomy [18], and monthly income [3]. Furthermore, among the individual-related factors, earlier studies have identified aspects such as educational level, age, nursing experience [6, 19–21], duration of service, job title, and type of job contract [9, 17, 18]. In an earlier Finnish study [18], several background variables were positively related to nurse engagement, including working as an assistant nurse manager, working in an outpatient clinic, having a temporary job contract, and having less than a year of work experience in the current organisation. Among nursing professionals, midwives were less engaged than other nurses, with 48% reporting disengagement [18].

In a study by Lepistö et al. [22], it was found that the engagement of healthcare professionals (72% representing nursing staff) in Finnish university hospitals was high. Generational differences in engagement were also observed, with a trend towards higher scores in older groups. However, only the baby boomers' dedication and absorption results differed significantly [22]. In contrast, Junttila et al. [18] found that only 9% of nurses in Finland were engaged, a lower percentage than reported in previous studies in the United States [23] and Italy [24] using the same instrument.

There are only a few studies on nurse engagement in Finland [18, 22, 25], and there has been no systematic national monitoring on the topic. However, since 2018, the Consortium for the National Benchmarking of Nursing-Sensitive Outcomes (later: Consortium) has annually conducted a survey on nurse engagement. In 2024, the Consortium had 15 member organisations covering 65% of Finnish public healthcare services [26]. The Consortium has emphasised the importance of monitoring long-term nurse engagement and related factors at the national level to identify potential trends and challenges in retaining nurses within the healthcare sector. Previous studies have not examined nurse engagement over multiple years.

## 2. Methods

### 2.1. Aim, Objective and Research Questions

The aim of this study is to describe and explain the level of nurse engagement in the period 2019–2022 and identify related background variables and drivers of nurse engagement in hospital settings. The objective is to provide knowledge that can support nurse managers and leaders in developing a more engaging work environment and effective leadership for nurses. The research questions are:

1. What were the levels of nurse engagement from 2019 to 2022?
2. How are the background variables related to nurse engagement levels?
3. How did the drivers of nurse engagement change between the years 2019 and 2022?
4. How are the drivers of nurse engagement related to the level of nurse engagement?

### 2.2. Design

This study employed an analytical cross-sectional multicentre design with a four-year time series (2019–2022). The STROBE checklist was used as the reporting guideline.

### 2.3. Study Setting and Sampling

The study setting included all five university hospitals and four central hospitals within the Consortium in Finland. University hospitals provide tertiary-level care and are affiliated with medical universities. Central hospitals also offer specialized care, but their services are not as extensive or advanced as those of university hospitals. A census method of collecting data was used, inviting all staff nurses, midwives, and assistant nurse managers (hereafter referred to as “nurses”) from all nine organisations to participate in the study.

### 2.4. Instrument

The original Nurse Engagement Survey (NES) contains 48 statements [2], and it was also used in Finland during the period 2015-2016 [25]. Later, the NES instrument was modified to ensure that it included all aspects relevant to accreditation for Magnet® status, as accreditation was the goal of some Finnish hospitals. The American Nurses Credentialing Center was consulted during the modification process so that the accreditation criteria were fulfilled. The modified NES (mNES) consists of 53 statements rated on a 6-point Likert scale (6 = totally agree, 5 = agree, 4 = somewhat agree, 3 = somewhat disagree, 2 = disagree, and 1 = totally disagree). There was no formal back translation. Instead, the survey was translated into Finnish by experts in nursing science and into Swedish by clinical, Swedish-speaking nurses and experts. In addition, the Finnish and Swedish surveys were piloted in 2015 with nearly 2000 nurses, and feedback on the survey's feasibility was collected.

One component of the mNES concerns personal engagement (4 statements), which is the key sum variable of this study (outcome). The level of nurse engagement is calculated according to the original algorithm: respondents were considered “engaged” if they answered at least “agree” to two of four statements and “strongly agree” to at least two of them [2, 25]. In addition, the mNES instrument contains eight sum variables, which are drivers of nurse engagement. The drivers of nurse engagement are fundamentals of quality nursing care (11 statements), leadership access and responsiveness (9 statements), autonomy (4 statements), interprofessional collaboration (4 statements), RN-to-RN collaboration (5 statements), professional development (5 statements), adequacy of resources and staffing (5 statements), and professional relevance (6 statements) (Table 1). The internal consistency of the mNES was tested by Cronbach's α coefficient. Satisfactory values [27] for Cronbach's α coefficient were achieved for the overall scale (0.91) and all sum variables (range 0.70–0.92).

Respondents' background information consisted of organisation type (university hospital or central hospital), unit type (outpatient care or inpatient care), job title (staff nurse, midwife, assistant nurse manager), type of employment (permanent or temporary), length of employment (under one year, 1–3 years, 4–6 years, 7–15 years, over 15 years), education (master's degree or higher from university, master's degree from university of applied sciences, bachelor's degree or diploma in nursing), and work shift (daytime work, two-shift work, three-shift work, i.e., alternating between morning, evening and night shifts and night work only).

### 2.5. Data Collection

Data were collected via electronic surveys in the years 2019, 2020, 2021, and 2022. Data collection typically occurred over a four-week period in spring, except in 2022, when it was postponed to autumn due to a nurses' strike. Direct distribution of the survey to the target group was not possible in all participating organisations due to the lack of comprehensive email lists. Depending on the organisation's method, the information letter and the link to the survey were either emailed directly to the employee's email or shared through a unit's nurse manager. Therefore, an exact response rate cannot be determined. In the study by Junttila et al. [18], also conducted in Finland, the imputed response rate—based on a comparison between the number of respondents and the number of hospital vacancies in the corresponding professional groups—varied between 7% and 37% across organisations in 2020, which can be considered an indicative annual rate for this study as well, given the similar data collection process and topic. Completing the survey took approximately 15 min, and respondents received two reminders during the response period.

### 2.6. Data Analysis

Respondents who had not answered at least 26 statements (around 50%) were excluded from the analysis. Categorical variables were summarised with counts and percentages, and continuous variables with means and standard deviations. The statistical analysis for studying associations between explanatory variables (background variables, drivers of engagement) with the level of nurse engagement and year of response was performed separately.

The associations between the level of nurse engagement or response year and background variables were studied one by one with a chi-square test. After the univariate approach, modelling was continued with multinomial logistic regression including all background variables. Odds ratios (OR) with 95% Wald confidence intervals (95% CI) are reported. The associations between the level of nurse engagement or response year and the drivers of engagement were studied with linear mixed models. Correlations between the drivers of engagement were performed with Pearson correlation, and Cronbach's α was calculated.

The normality of variables was evaluated visually and tested with the Shapiro–Wilk test. All tests were performed as two-sided with the significance level set at 0.05. Analyses were carried out using SAS version 9.4 for Windows (SAS Institute Inc., Cary, NC, USA).

## 3. Results

### 3.1. Characteristics of the Sample

In 2019, the study included five university hospitals and one central hospital, with a total of 7207 participants. In subsequent years, four central hospitals joined the five university hospitals in the study, resulting in 4293 participants in 2020, 6701 in 2021, and 6452 in 2022. The total number of respondents was 24,653. The majority of respondents worked in inpatient care (55.0%), at university hospitals (86.1%), as staff nurses (88.2%), with permanent contracts (81.9%), and in three-shift work (52.9%). Most respondents had a bachelor's degree (80.2%) and over 15 years of employment (31.5%) (Table 2).

### 3.2. Level of Nurse Engagement 2019–2022

**Figure 1:**
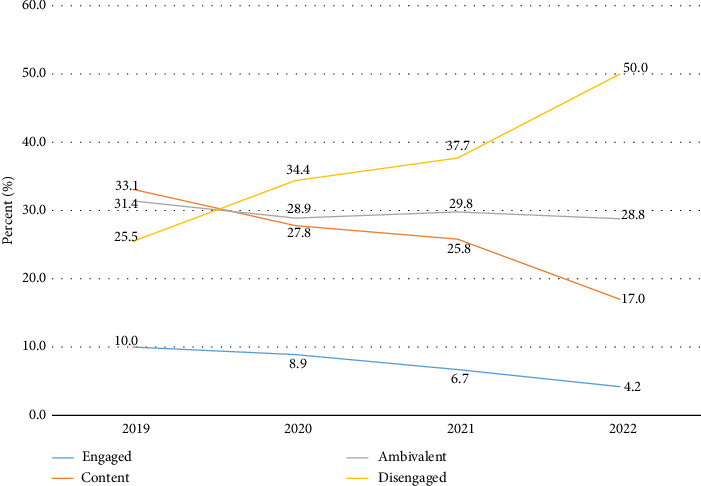
Level of nurse engagement in 2019–2022 (*n*=24,647). Chi-square test: Differences in the level of nurse engagement were statistically significant between years (*p* < 0.0001).

Nurse engagement varied over the study period, with 10.0%–4.2% of nurses being engaged, 33.1%–17.0% content, 31.4%–28.8% ambivalent, and 25.5%–50.0% disengaged. The number of disengaged nurses increased, while the number of content and engaged nurses decreased across years. The number of ambivalent nurses remained fairly stable, with some fluctuation over the years. Differences in the level of nurse engagement were statistically significant between years (*p* < 0.0001) (Figure 1).

### 3.3. Relationship Between Background Variables and the Level of Nurse Engagement

In univariate analysis, all background variables were statistically significantly (*p* < 0.0001) in relation to the level of nurse engagement. Nurses working in university hospitals (7.4%) and nurses with temporary contracts (10.3%) were more engaged than nurses working in central hospitals (7.0%) or with permanent contracts (6.7%). Assistant nurse managers (14.9%) were more engaged than midwives (4.4%) and staff nurses (7.2%). Nurses with master's degrees from a university of applied sciences (9.2%) were more engaged than those with other educational backgrounds (6.2%–7.6%). Additionally, nurses working in outpatient care (8.0%) and on day time (9.8%) were more engaged than nurses working in inpatient care (6.9%) and on shift work (6.0%–8.0%). Nurses with less than 1 year of working experience (12.7%) were more engaged than nurses with over 1 year of experience (4.8%–7.9%). Midwives, nurses working three shifts, and nurses with 4–6 years of employment were the least engaged among the respondents (Table 3).

A multinomial logistic regression model was performed to evaluate the relationship between the level of nurse engagement and all background variables, including years. All background variables remained statistically significant, as in the univariate approach. The results are reported in Table 4.

### 3.4. Variation of Drivers of Nurse Engagement Between the Years 2019 and 2022

The lowest mean scores for drivers of nurse engagement were found for leadership access and responsiveness (range 3.62–3.77), and the highest were found for professional relevance (range 4.59–4.75). The mean scores for each driver of nurse engagement varied by 0.01–0.21 decimal points over the period or remained at the same level. For six drivers, there were statistically significant differences between the years (Table 5).

### 3.5. Relationship Between the Drivers of Engagement and the Level of Nurse Engagement

**Figure 2:**
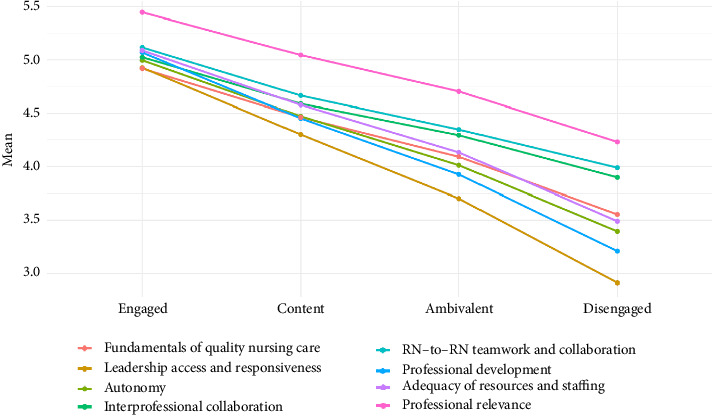
Relation of drivers to level of nurse engagement.

All drivers of nurse engagement were statistically significant (*p* < 0.0001) in relation to the level of nurse engagement. The mean scores for these drivers ranged from 4.92 to 5.44 for engaged nurses and from 2.91 to 4.23 for disengaged nurses (Figure 2, Table 6).

## 4. Discussion

### 4.1. Discussion of the Results

The aim of this study was to describe and explain the level of nurse engagement in the period 2019–2022 and identify related background variables and drivers of nurse engagement in hospital settings. Previous studies have not examined nurse engagement over multiple years. The objective here was to provide knowledge that can support nurse managers and leaders in developing a more engaging work environment and effective leadership for nurses. Based on the results of this study, the number of disengaged nurses increased, while the number of engaged and content nurses decreased over the period. However, the number of ambivalent nurses remained stable despite some variation over the years. In this study, the proportion of engaged nurses was clearly lower than in studies conducted in the United States [23] and Italy [24], which also used the NES instrument. In the above-mentioned studies, data collection occurred before the COVID-19 pandemic. Similarly, part of the data for this study was collected in 2019, also before the pandemic. However, in Finland that year, 25.5% of nurses were classified as disengaged, a significantly higher proportion than the 4% reported in the United States [23], the 13% reported in Italy [24], and the 13% reported in a previous Finnish study with data collected in 2015 and 2016 [25]. The Finnish healthcare system has long had problems with staff shortages, which has put a strain on nurses. Excessive workloads, long shifts, and inadequate staffing levels may have affected nurses' motivation and engagement.

The results of this study are alarming considering the attractiveness of the nursing profession and retention of nurses. The COVID-19 pandemic may have affected results from 2020 onwards, as the health crisis created personal and professional challenges for nurses. In the study of Penturij-Kloks et al. [28], nurse engagement decreased significantly after the COVID-19 outbreak, but the decline stabilised partly during follow-up. This study shows the opposite results over a longer period of time and does not show either a stabilisation or increase in engagement. The results of this study may have also been influenced by the nurses' strike in 2022, which affected pay and working conditions, and by chronic labour shortages, which may have increased the workload of nurses. The quantity of work and resources has been shown to directly affect nurses' work engagement [6, 21], while monthly income is also linked to their engagement [3]. To keep nurses engaged, nurse managers and leaders should keep patient care manageable [11, 21]. They could also offer other tasks that match nurses' interests and development and career opportunities, such as research and development and administrative tasks, which could help avoid overload and promote engagement, as also noted by Pericak et al. [6]. While the current workforce is strained by workforce shortages, managers must strive to improve the attractiveness of organisations [28]. This creates challenges for nurse leaders and requires cooperation and innovations at different levels of multidisciplinary management.

This study shows that nurse engagement and disengagement varied from year to year over the studied period. Previous research has shown that work engagement is a highly stable phenomenon; feelings of work engagement are quite permanent and long-lasting [14]. However, the persistence of work engagement seems to depend to some extent on the time horizon over which it is measured. Shorter time spans, from a few days to a few weeks, have shown that work engagement varies [15], but this is influenced by the support and feedback of nurse managers and leaders [17]. The data in this study do not offer an explanation for the alarming trend regarding nurse engagement and disengagement. Further research is therefore needed to analyse whether the decline in engagement is set to continue and how it could be prevented.

In this study, the respondents' background variables were related to the level of nurse engagement. Nurses working in university hospitals and nurses with temporary contracts were more engaged than nurses working in central hospitals or with permanent contracts. This result is consistent with the findings of Junttila et al. [18], and it may be explained by the fact that a temporary contract can be a route to permanent employment or career development, which motivates nurses. A temporary contract may also better fit some nurses' life situations, such as balancing studies or family, making it a positive and flexible employment option. Our study also found that assistant nurse managers were more engaged compared to midwives and staff nurses, which is in line with the results of Mustonen et al. [25]. Furthermore, this result is also consistent with the findings of Al-Ahmari and Kattan [19] and Gao et al. [17], who reported that nursing staff in managerial positions demonstrated greater engagement than general nursing staff. In addition, the results of this study show that nurses with a master's degree from a university of applied sciences were more engaged than nurses with other educational backgrounds. Higher education can enable more responsible and independent work and lead to better career development opportunities, avoiding stress and increasing engagement [6]. In contrast, other studies [17, 19, 21] have not found that different educational levels affect nurses' work engagement.

This study shows that nurses working in outpatient care and on day shift were more engaged than nurses working in inpatient care and on shift work, which differs from the findings of Keyko et al. [21]. We can only speculate about the reasons for this. It may be that day shifts offer a more predictable schedule, allowing nurses to maintain a better natural work–life balance and circadian rhythm than shift work. In addition, outpatient care may be less stressful than inpatient care, where nurses care for critically ill patients and emergencies. In a study by Junttila et al. [18], nurse engagement was higher in early- and late-career nurses, and the results of this study are similar. This may be because early-career nurses are driven by enthusiasm and growth and receive more structured support, such as mentoring programmes or induction, while late-career nurses feel more confident in their own skills and expertise, which increases job satisfaction and engagement. The findings of Pericak et al. [6] support this reasoning, as a higher ability to cope correlates with higher engagement. In contrast, mid-career nurses may face challenges such as job fatigue, stagnation, and competing personal responsibilities, which can undermine engagement. It is therefore important that nurse leaders promote nurse engagement throughout their careers and recognise nurses of all generations [18, 24].

This study shows that the mean scores of the drivers of nurse engagement did not change much or remained at approximately the same level between years. Each year, the lowest mean scores were found in leadership access and responsiveness, and it was found that disengaged nurses in particular gave low scores. As the number of disengaged nurses increased during the study period, it is possible that nurses may have expected more support and stronger leadership from managers due to a simultaneous health crisis and staff shortage. The results of this study show that all drivers of nurse engagement were related to the level of nurse engagement, and, on average, they were more strongly associated with engaged nurses than with disengaged nurses. Therefore, all of these factors affecting nurse engagement must be taken into account by management and leadership. The results of this study align with those of previous studies that have identified organisational support and climate [10], professional resources [4], regular feedback and recognition [18], and rewards and structural empowerment [20] as factors related to nurse engagement. Therefore, nurse leaders and managers must adopt a proactive, adaptive, and personal approach to leadership. By responding to workload, changing expectations, and external challenges, and by recognising individual contributions, leaders can promote nurse engagement and meet the changing needs of the workforce. Management practices that promote nurse engagement are important, as nurse engagement is a key factor in staff retainment and lower turnover rates [13, 29] and also in providing quality patient care and producing better patient outcomes [30]. Healthcare organisations should regularly assess the engagement levels of their nursing workforce and create and prioritise leadership strategies designed to attract, retain, and improve nurse engagement.

### 4.2. Strengths and Limitations

The strength of this study is the large, nationwide dataset and the 4-year time span, which provide unique insights into the phenomenon under study. One limitation of the study is that the response activity could not be accurately determined. The response activity was likely influenced by the COVID-19 pandemic and nurses' strike. Additionally, a dropout analysis was not possible.

The NES instrument has been developed in the context of nursing based on literature and factor analysis [2]. Here, we evaluated the reliability of this instrument as satisfactory, as in earlier studies [24, 25]. However, the psychometric properties of the mNES instrument should be further tested. While this study was conducted in Finland, its findings may offer valuable insights for other developed countries experiencing similar challenges with nurse engagement in today's dynamic and unpredictable healthcare environment.

### 4.3. Recommendations for Further Research

Intervention studies are needed to promote nurse engagement in hospital settings. However, long-term follow-up studies are required to comprehensively monitor changes in nurse engagement at the national level and to facilitate comparisons between different countries. It remains to be determined whether this stabilisation marks the beginning of a recovery in work engagement or indicates a permanent reduction.

## 5. Conclusions

This study produced novel results on levels of nurse engagement and related factors in a hospital setting, using unique, national-level data collected over four years (2019–2022). The findings revealed an increase in disengaged nurses and a decrease in engaged and content nurses, while the number of ambivalent nurses remained stable. The results are worrying considering the attractiveness of the nursing profession and retention of nurses. The study provides insights and guidance for nurse leaders and managers in creating more engaging work environments and improving leadership practices. The results should also contribute to developing targeted interventions to enhance nurse engagement in hospitals. To our knowledge, this is the first study to examine nurse engagement for a national-level sample over multiple years.

## Figures and Tables

**Table 1:** Sum variables of the modified nurse engagement survey.

Sum variable	Statements (n)	Description
Fundamentals of quality nursing care	11	Vision and values of the organisation, competence of co-workers, realization of evidence-based nursing practice, nursing quality, occupational safety and well-being
Leadership access and responsiveness	9	The role and actions of immediate supervisor, visibility of the highest nurse leader, recognition, feedback
Autonomy	4	Input into patients' medical care planning and treatment, level of independence, participation in decision-making affecting one's work, respect to nurses' contribution
Interprofessional collaboration	4	Collaboration with physicians, abuse behaviour, ethical courage relating to physicians' actions
RN-to-RN collaboration	5	Ethical courage relating to nurses' actions, proactive help to others, access to clinical experts, resolving of conflicts, nurses' active role in improving patient care
Professional development	5	Career path, professional growth, support to further/additional education, knowledge of own role escalation
Adequacy of resources and staffing	5	Access to necessary equipment and supplies, new technology in supporting nursing, manageable workload, enough time with patients, compromised patient care
Professional relevance	6	Relationships with other nurses and support staff, flexibility in work shifts, meaningful connections with patients and/or their families, pride of one's profession
Personal engagement	4	Inspiration, motivation, commitment

**Table 2:** Background of the respondents (*n* = 24,653).

Background variables	Year				Total
	2019	2020	2021	2022	
	*n* = 7207	*n* = 4293	*n* = 6701	*n* = 6542	*n* = 24,653
	*n* (%)	*n* (%)	*n* (%)	*n* (%)	*n* (%)
Organisation type					
University hospital	6832 (94.8)	3648 (85.0)	5509 (82.2)	5240 (81.2)	21,229 (86.1)
Central hospital	375 (5.2)	645 (15.0)	1192 (17.8)	1212 (18.8)	3424 (13.9)
Job title					
Assistant nurse manager	340 (4.7)	197 (4.6)	326 (4.9)	313 (4.9)	1176 (4.8)
Midwife	555 (7.7)	311 (7.2)	381 (5.7)	486 (7.5)	1733 (7.0)
Staff nurse	6312 (87.6)	3785 (88.2)	5994 (89.4)	5653 (87.6)	21,744 (88.2)
Type of employment					
Permanent	5603 (78.3)	3440 (80.8)	5503 (82.3)	5522 (86.1)	20,068 (81.9)
Temporary	1553 (21.7)	819 (19.2)	1182 (17.7)	890 (13.9)	4444 (18.1)
Education					
Master's degree or higher (university)	209 (2.9)	101 (2.4)	164 (2.4)	170 (2.6)	644 (2.6)
Master's degree (university of applied sciences)	336 (4.7)	235 (5.5)	328 (4.9)	344 (5.3)	1243 (5.0)
Bachelor's degree	6559 (92.3)	3924 (91.6)	4625 (69.1)	4551 (70.7)	19,659 (80.2)
Diploma in nursing	0 (0.0)	24 (0.6)	1579 (23.6)	1376 (21.4)	2979 (12.2)
Work shift					
Daytime work	2170 (30.3)	1373 (32.1)	2065 (30.9)	2078 (32.3)	7686 (31.3)
2-shift work	964 (13.4)	569 (13.3)	914 (13.7)	952 (14.8)	3399 (13.8)
3-shift work	3975 (55.4)	1975 (46.2)	3685 (55.1)	3358 (52.1)	12,993 (52.9)
Only night work	64 (0.9)	356 (8.3)	30 (0.5)	53 (0.8)	501 (2.0)
Length of employment					
Under 1 year	778 (10.8)	295 (6.9)	494 (7.4)	472 (7.3)	2039 (8.3)
1–3 years	1123 (15.6)	796 (18.6)	1184 (17.7)	1096 (17.0)	4199 (17.1)
4–6 years	1132 (15.7)	569 (13.3)	987 (14.8)	1014 (15.8)	3702 (15.0)
7–15 years	2389 (33.2)	1161 (27.1)	1733 (25.9)	1639 (25.5)	6922 (28.1)
Over 15 years	1780 (24.7)	1465 (34.2)	2294 (34.3)	2213 (34.4)	7752 (31.5)
Work unit					
Outpatient care	2771 (40.8)	1954 (46.5)	3069 (46.1)	3042 (47.4)	10,836 (45.0)
Inpatient care	4021 (59.2)	2245 (53.5)	3587 (53.9)	3373 (52.6)	13,226 (55.0)

**Table 3:** Relationship between background variables and the level of nurse engagement (*n* = 24,506).

Background variable	Level of nurse engagement				*p* value^1^
	Engaged	Content	Ambivalent	Disengaged	
	*n* (%)	*n* (%)	*n* (%)	*n* (%)	
Year					< 0.0001
2019	722 (10.0)	2383 (33.1)	2264 (31.4)	1838 (25.5)	
2020	381 (8.9)	1192 (27.8)	1243 (28.9)	1477 (34.4)	
2021	447 (6.7)	1727 (25.8)	1999 (29.8)	2526 (37.7)	
2022	271 (4.2)	1096 (17.0)	1856 (28.8)	3225 (50.0)	
Organization type					< 0.0001
University hospital	1581 (7.4)	5436 (25.6)	6240 (29.4)	7972 (37.6)	
Central hospital	240 (7.0)	962 (28.2)	1122 (32.8)	1094 (32.0)	
Job title					< 0.0001
Assistant nurse manager	175 (14.9)	497 (42.3)	323 (27.5)	181 (15.4)	
Midwife	76 (4.4)	344 (19.9)	509 (29.4)	803 (46.4)	
Staff nurse	1570 (7.2)	5557 (25.6)	6530 (30.0)	8082 (37.2)	
Type of employment					< 0.0001
Permanent	1353 (6.7)	5065 (25.3)	6014 (30.0)	7630 (38.0)	
Temporary	459 (10.3)	1311 (29.5)	1301 (29.3)	1373 (30.9)	
Education					0.0152
Master's degree or higher (university)	40 (6.2)	181 (28.1)	201 (31.2)	222 (34.5)	
Master's degree (university of applied sciences)	114 (9.2)	331 (26.6)	370 (29.8)	428 (34.4)	
Bachelor's degree	1426 (7.3)	5089 (25.9)	5808 (29.5)	7331 (37.3)	
Diploma in nursing	225 (7.6)	761 (25.5)	949 (31.9)	1043 (35.0)	
Work shift					< 0.0001
Daytime work	750 (9.8)	2327 (30.3)	2322 (30.2)	2285 (29.7)	
2-shift work	247 (7.3)	922 (27.1)	1013 (29.8)	1217 (35.8)	
3-shift work	776 (6.0)	2976 (22.9)	3865 (29.8)	5372 (41.4)	
Only night work	40 (8.0)	157 (31.3)	139 (27.7)	165 (32.9)	
Length of employment					< 0.0001
Under 1 year	259 (12.7)	691 (33.9)	603 (29.6)	486 (23.8)	
1–3 years	321 (7.6)	1111 (26.5)	1217 (29.0)	1549 (36.9)	
4–6 years	177 (4.8)	794 (21.4)	1053 (28.5)	1677 (45.3)	
7–15 years	445 (6.4)	1668 (24.1)	2106 (30.4)	2700 (39.0)	
Over 15 years	614 (7.9)	2132 (27.5)	2368 (30.6)	2637 (34.0)	
Work unit					0.0015
Outpatient care	862 (8.0)	2862 (26.4)	3176 (29.3)	3934 (36.3)	
Inpatient care	910 (6.9)	3367 (25.5)	3954 (29.9)	4991 (37.7)	

^1^Chi-square test.

**Table 4:** A model for the association between the level of nurse engagement and background variables.

Background variable	Level of nurse engagement					
	Content	*p* value	Ambivalent	*p* value	Disengaged	*p* value
	OR [95% CI]^1^		OR [95% CI]^1^		OR [95% CI]^1^	
Year						
2019	0.76 [0.64–0.90]	0.0018^∗^	0.40 [0.34–0.47]	< 0.0001^∗^	0.17 [0.14–0.20]	< 0.0001^∗^
2020	0.71 [0.59–0.86]	0.0004^∗^	0.44 [0.36–0.53]	< 0.0001^∗^	0.27 [0.22–0.32]	< 0.0001^∗^
2021	0.95 [0.80–1.12]	0.5249	0.65 [0.55–0.76]	< 0.0001^∗^	0.46 [0.39–0.54]	< 0.0001^∗^
2022	1.00 (reference)					
Organization type						
University hospital	1.00 (reference)					
Central hospital	1.16 [0.99–1.36]	0.0743	1.04 [0.89–1.23]	0.5991	0.66 [0.57–0.78]	< 0.0001^∗^
Job title						
Assistant nurse manager	0.81 [0.65–0.99]	0.0413^∗^	0.46 [0.37–0.58]	< 0.0001^∗^	0.23 [0.18–0.30]	< 0.0001^∗^
Midwife	1.34 [1.03–1.75]	0.0286^∗^	1.67 [1.29–2.16]	0.0001^∗^	2.18 [1.69–2.82]	< 0.0001^∗^
Staff nurse	1.0 (reference)					
Type of employment						
Permanent	1.00 (reference)					
Temporary	0.82 [0.70–0.96]	0.0114^∗^	0.73 [0.62–0.85]	< 0.0001^∗^	0.65 [0.56–0.76]	< 0.0001^∗^
Education						
Master's degree or higher (university)	1.00 (reference)					
Master's degree (university of applied sciences)	0.67 [0.44–1.01]	0.0570	0.63 [0.42–0.96]	0.0296^∗^	0.62 [0.41–0.95]	0.0264^∗^
Bachelor's degree	0.76 [0.53–1.09]	0.1321	0.65 [0.45–0.93]	0.0182^∗^	0.65 [0.45–0.93]	0.0183^∗^
Diploma in nursing	0.59 [0.40–0.88]	< 0.0001^∗^	0.48 [0.32–0.72]	0.0003^∗^	0.36 [0.24–0.54]	< 0.0001^∗^
Work shift						
Daytime work	1.00 (reference)					
2-shift work	1.19 [1.00–1.42]	0.0500^∗^	1.31 [1.10–1.56]	0.0024^∗^	1.70 [1.42–2.02]	< 0.0001^∗^
3-shift work	1.16 [0.99–1.35]	0.0534	1.54 [1.32–1.79]	< 0.0001^∗^	2.26 [1.95–2.63]	< 0.0001^∗^
Only night work	1.32 [0.90–1.92]	0.1568	1.18 [0.80–1.74]	0.3995	1.49 [1.02–2.18]	0.0414^∗^
Length of employment						
Under 1 year	0.78 [0.63–0.96]	0.0181^∗^	0.63 [0.51–0.78]	< 0.0001^∗^	0.45 [0.37–0.56]	< 0.0001^∗^
1–3 years	0.99 [0.82–1.18]	0.8752	0.94 [0.78–1.13]	0.5183	0.99 [0.82–1.18]	0.9463
4–6 years	1.24 [1.02–1.52]	0.0333^∗^	1.41 [1.16–1.72]	0.0007^∗^	1.92 [1.58–2.33]	< 0.0001^∗^
7–15 years	1.04 [0.90–1.21]	0.5878	1.16 [1.00–1.35]	0.0441^∗^	1.32 [1.14–1.52]	0.0003^∗^
Over 15 years	1.00 (reference)					
Work unit						
Outpatient care	0.98 [0.85–1.11]	0.7072	1.04 [0.91–1.19]	0.5555	1.24 [1.09–1.41]	0.0019^∗^
Inpatient care	1.00 (reference)					

*Note:* The reference class of dependent variable is “Engaged” and reference classes for background variables are shown in the table.

^1^A multinomial logistic regression model. Odds ratios (OR) with 95% confidence intervals and *p* values are reported (^∗^ = statistically significant *p* values at a significance level of 0.05).

**Table 5:** Drivers of nurse engagement in 2019–2022.

Drivers of nurse engagement^1^	Year					*p* value^2^
	2019	2020	2021	2022	Total	
	Mean (SD)	Mean (SD)	Mean (SD)	Mean (SD)	Mean (SD)	
Fundamentals of quality nursing care	4.10 (0.69)	4.09 (0.71)	4.06 (0.71)	3.97 (0.73)	4.05 (0.71)	< 0.0001
Leadership access and responsiveness	3.75 (1.10)	3.62 (1.10)	3.62 (1.07)	3.63 (1.06)	3.66 (1.08)	< 0.0001
Autonomy	4.07 (0.91)	4.00 (0.93)	3.94 (0.93)	3.89 (0.92)	3.98 (0.93)	< 0.0001
Interprofessional collaboration	4.29 (0.92)	4.29 (0.95)	4.26 (0.95)	4.28 (0.95)	4.28 (0.94)	0.0884
RN-to-RN teamwork and collaboration	4.36 (0.81)	4.36 (0.85)	4.34 (0.85)	4.36 (0.85)	4.36 (0.84)	0.5444
Professional development	3.99 (0.97)	3.88 (1.01)	3.87 (1.01)	3.78 (1.04)	3.88 (1.01)	< 0.0001
Adequacy of resources and staffing	4.16 (0.93)	4.12 (0.95)	4.09 (0.96)	3.97 (1.01)	4.08 (0.97)	< 0.0001
Professional relevance	4.75 (0.64)	4.69 (0.68)	4.66 (0.70)	4.59 (0.70)	4.67 (0.68)	< 0.0001

Abbreviation: SD, standard deviation.

^1^Scale: 1–6 (1 = strongly disagree, 2 = disagree, 3 = tend to disagree, 4 = tend to agree, 5 = agree, and 6 = strongly agree).

^2^Linear mixed model.

**Table 6:** Relationship between the drivers of nurse engagement and the level of nurse engagement.

Drivers of nurse engagement^1^	Level of nurse engagement				
	Engaged	Content	Ambivalent	Disengaged	*p* value^2^
	Mean (SD)	Mean (SD)	Mean (SD)	Mean (SD)	
Fundamentals of quality nursing care	4.92 (0.50)	4.46 (0.50)	4.09 (0.53)	3.55 (0.64)	< 0.0001
Leadership access and responsiveness	4.93 (0.71)	4.30 (0.80)	3.70 (0.83)	2.91 (0.95)	< 0.0001
Autonomy	5.00 (0.67)	4.47 (0.70)	4.01 (0.75)	3.40 (0.86)	< 0.0001
Interprofessional collaboration	5.02 (0.74)	4.59 (0.77)	4.29 (0.83)	3.90 (1.00)	< 0.0001
RN-to-RN teamwork and collaboration	5.12 (0.65)	4.67 (0.66)	4.35 (0.71)	3.99 (0.89)	< 0.0001
Professional development	5.07 (0.65)	4.45 (0.71)	3.93 (0.76)	3.21 (0.94)	< 0.0001
Adequacy of resources and staffing	5.09 (0.63)	4.58 (0.68)	4.13 (0.76)	3.49 (0.97)	< 0.0001
Professional relevance	5.44 (0.38)	5.04 (0.43)	4.70 (0.50)	4.23 (0.70)	< 0.0001

^1^Scale 1–6 (1 = strongly disagree, 2 = disagree, 3 = tend to disagree, 4 = tend to agree, 5 = agree, and 6 = strongly agree).

^2^Linear mixed model.

## Data Availability

The data that support the findings of this study are available from the corresponding author upon reasonable request.
